# Jejunogastric Intussusception as a Delayed Complication Following Laparoscopic Pancreatoduodenectomy

**DOI:** 10.7759/cureus.82914

**Published:** 2025-04-24

**Authors:** Camila Sotomayor, Alan Camhi, Claudio Barrientos, Mauricio Gabrielli, Nicolás Jarufe, Jorge A Martinez

**Affiliations:** 1 Department of Hepatobiliary and Pancreatic Surgery, Pontificia Universidad Católica de Chile, Santiago, CHL; 2 Department of Oesophagogastric Surgery, Pontificia Universidad Católica de Chile, Santiago, CHL

**Keywords:** gastrointestinal surgery, jejunogastric intussusception, laparoscopic surgery, surgical oncology, whipple procedure complication

## Abstract

Jejunogastric intussusception is a rare yet potentially serious complication following gastric surgery. Its occurrence after pancreaticoduodenectomy (Whipple procedure) is infrequently documented in the literature. Here, we present the case of a 41-year-old patient who developed jejunogastric intussusception three years following pancreaticoduodenectomy performed for a neuroendocrine tumor. The diagnosis was confirmed by computed tomography and upper endoscopy. The condition was successfully managed via exploratory laparoscopy. To our knowledge, this report describes the first case of jejunogastric intussusception following laparoscopic pancreaticoduodenectomy, highlighting successful management using the same minimally invasive approach. A review of the relevant literature is included to contextualize this uncommon complication.

## Introduction

Pancreaticoduodenectomy is a technically demanding procedure primarily performed for the oncological resection of periampullary tumors. This surgery involves the removal of the pancreatic head, duodenum, proximal jejunum, and, frequently, the gastric antrum. A variant known as pylorus-preserving pancreaticoduodenectomy conserves the gastric antrum and proximal duodenum [1].

Common complications following pancreaticoduodenectomy include delayed gastric emptying, pancreatic fistula formation, postoperative hemorrhage, and complications related to vascular reconstruction [2]. Potential long-term complications encompass biliary strictures, small bowel obstruction, and peptic ulcers at anastomotic sites [3]. Laparoscopic pancreaticoduodenectomy reportedly has a complication profile comparable to that of open surgery [4].

Jejunogastric intussusception is an exceedingly rare complication, typically associated with gastric surgeries such as gastrectomy or bariatric procedures [5]. Nonetheless, the exact pathogenesis of jejunogastric intussusception remains unclear. The most widely proposed mechanism suggests that abnormal antegrade or retrograde peristalsis causes the afferent or efferent jejunal loop to invaginate into the stomach. Additional factors that may contribute include diminished gastric tone, enhanced retrograde peristalsis, and variations in the diameter of the anastomotic site; however, these hypotheses have not been definitively proven.

This case highlights an unusual presentation of jejunogastric intussusception following laparoscopic pancreaticoduodenectomy, thereby contributing to the limited existing literature on this subject.

## Case presentation

A 41-year-old woman presented to the emergency department with acute epigastric pain radiating to the left hypochondrium and back, accompanied by frequent episodes of bilious vomiting. Her medical history was notable for a laparoscopic pancreaticoduodenectomy performed three years earlier for a non-functional neuroendocrine tumor.

On examination, the patient was well-perfused, anicteric, and exhibited marked tenderness in the epigastric and left hypochondriac regions. Initial investigations included laboratory tests (Table 1), among which hyponatremia and leukocytosis stood out, a contrast-enhanced abdominal computed tomography (CT), and upper digestive endoscopy.

**Table 1:** Laboratory findings. AST: aspartate transaminase; LDH: lactate dehydrogenase; BUN: blood urea nitrogen

Test	Results	Reference range
C-reactive protein	0.41 mg/dL	Less than 0.5 mg/dL
Hemoglobin	16.1 g/dL	12.0–16.0 g/dL
Hematocrit	49.3%	36.0%–46.0%
White blood cells	11.8 × 10^3^/L	4.5-11.0 × 10^3^/L
Blood creatinine	1.19 mg/dL	0.5–0.9 mg/dL
AST	21 U/L	9–25 U/L
LDH	169 U/L	135–224 U/L
Total bilirubin	0.71 mg/dL	0–1 mg/dL
Blood proteins	7.4 g/dL	6–8 g/dL
Albumin	4.7 g/dL	3.5–5 g/dL
Sodium	128 mEq/L	135–145 mEq/L
Potassium	4.2 mEq/L	3.5–5 mEq/L
Chloride	104 mEq/L	100–108 mEq/L
Lipase	33 U/L	Less than 60 U/L
BUN	17 mg/dL	8–25 mg/dL

**Figure 1:**
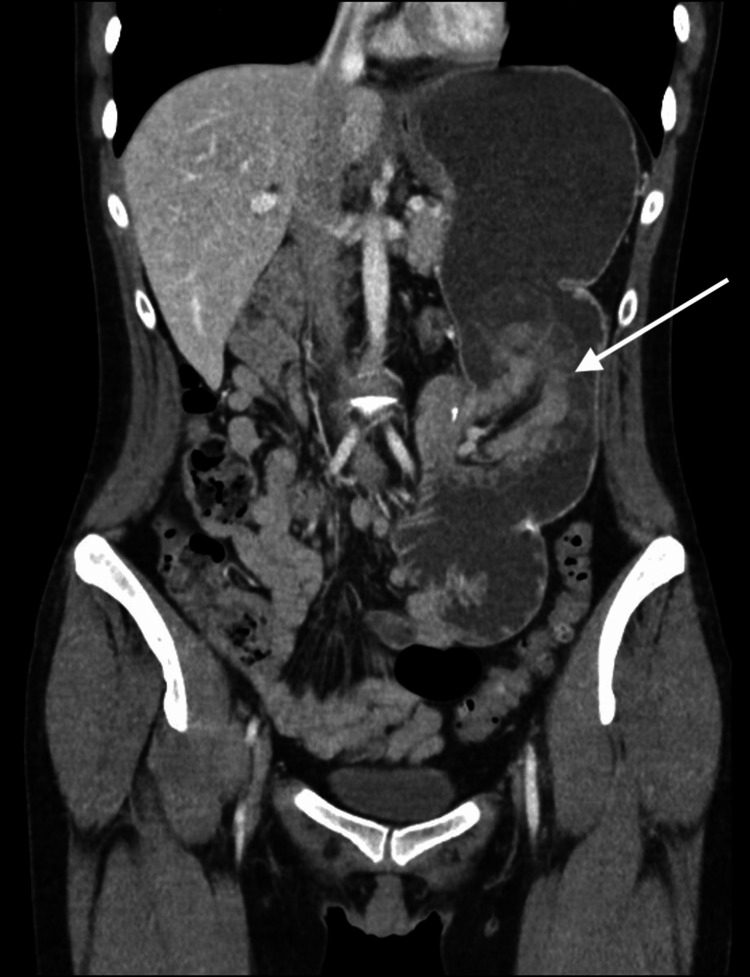
CT scan (coronal plane). Findings consistent with jejunal loop intussusception at the gastrojejunal anastomosis. Arrow: the examination reveals jejunal loop intussusception at the gastrojejunal anastomosis, resulting in marked dilation of the gastric chamber, without evidence of transmural ischemia of the intussuscepted loop.

**Figure 2:**
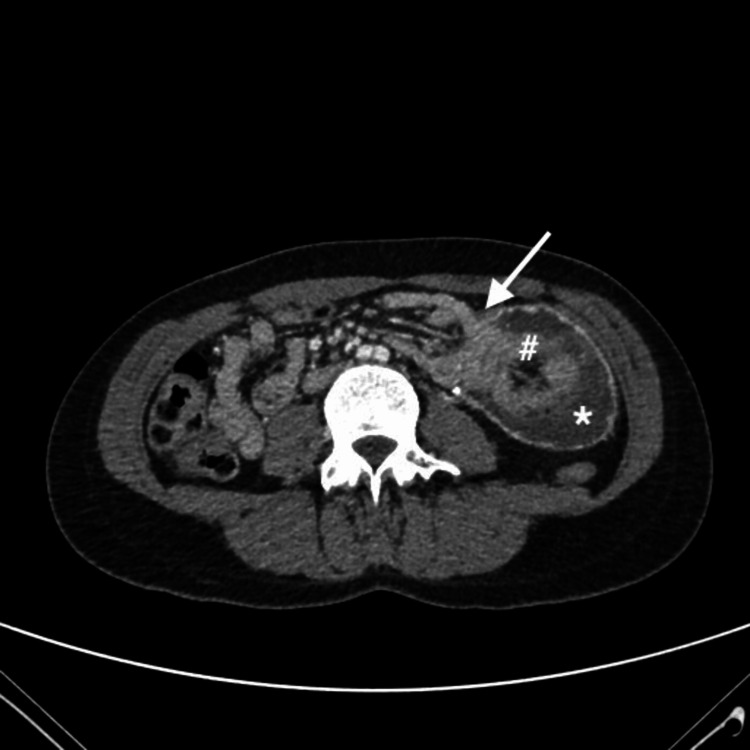
CT scan (axial plane). Findings consistent with jejunal loop intussusception at the gastrojejunal anastomosis. Arrow: the point of jejunal loop intussusception at the gastrojejunal anastomosis can be clearly identified; *: gastric chamber; #: jejunum.

The CT scan revealed jejunal loop intussusception at the site of the gastrojejunal anastomosis (Figures 1, 2).

**Figure 3:**
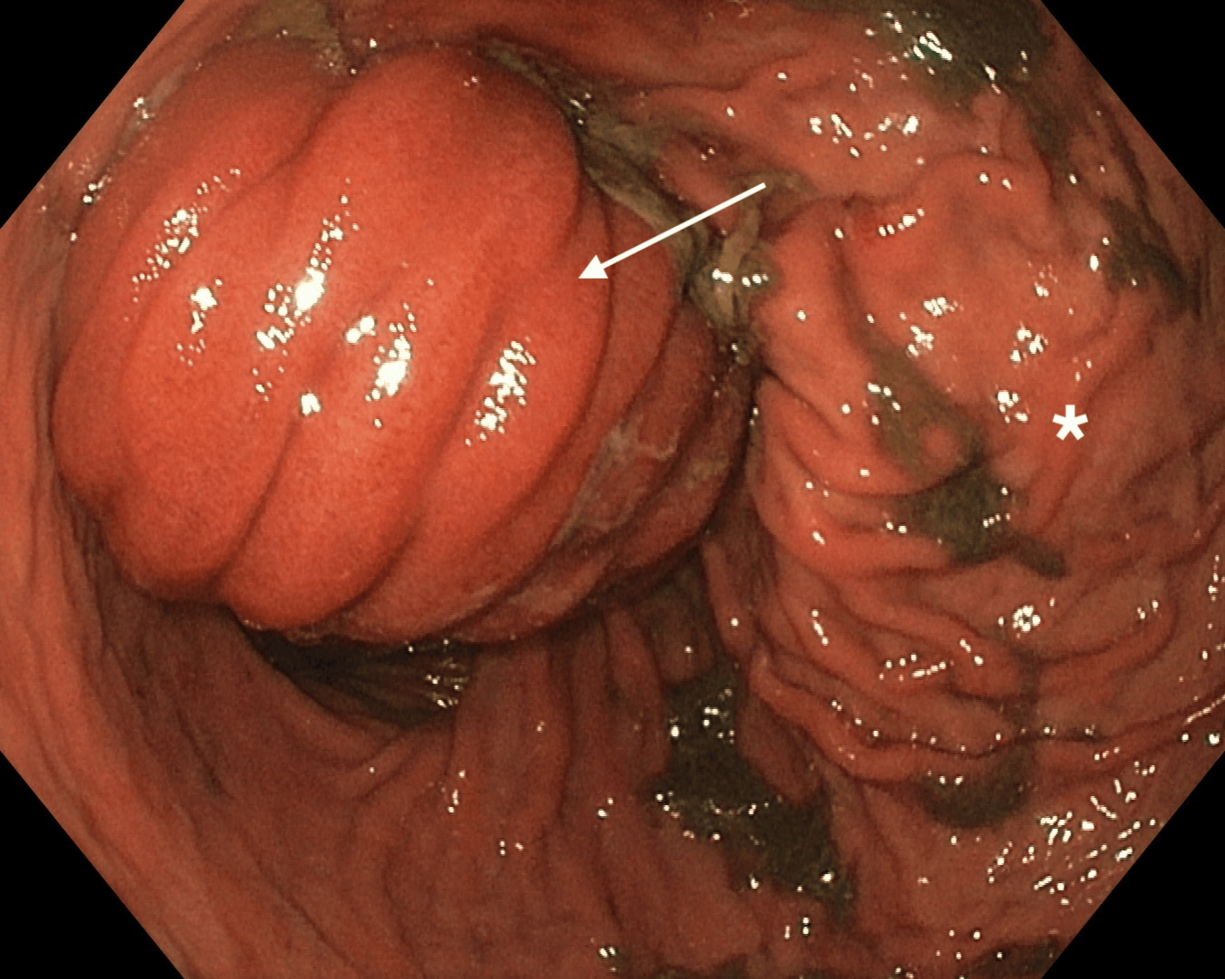
Upper endoscopy. The gastric chamber is wide and distensible, with abundant retained content that is almost completely aspirated. Retroflexed inspection of the subcardial region reveals a normal appearance without lesions. The mucosa of the fundus and body shows preserved folds. A small bowel loop is identified, occupying the entire distal lumen and extending toward the proximal body, with an intragastric length of approximately 7-8 cm. The loop appears viable, although a slightly violaceous area is noted where it contacts the greater curvature of the stomach. Intussusception could not be reduced with insufflation. Arrow: jejunal loop intussusception at the gastrojejunal anastomosis; *: gastric chamber.

Upper endoscopy confirmed that an 8 cm segment of the jejunum had intussuscepted into the stomach, while remaining viable (Figure 3).

Despite initial attempts at reduction through insufflation and mechanical manipulation, the intussusception persisted. Given the potential risks associated with continued manipulation, surgical intervention was deemed the most appropriate course of action. Laparoscopic surgery was performed, successfully reducing the intussusception and restoring the anatomy and function of the gastrointestinal tract. The jejunal segment was not resected because it was found to be viable. The patient’s postoperative course was uneventful, and she was discharged in stable condition.

## Discussion

Jejunogastric intussusception is a rare but potentially life-threatening condition, with mortality rates reaching up to 50% if left untreated within 48 hours [4]. Although its exact etiology remains unclear, contributing factors may include tumors or postoperative anatomical alterations. This diagnosis should be considered in patients with a history of gastric or pancreatic surgery who present with acute abdominal pain and vomiting.

Management typically requires surgical intervention involving reduction, resection, and reconstruction of the anastomosis, usually via exploratory laparotomy. While endoscopic reduction has been described, it is associated with a higher risk of recurrence [6-9].

To our knowledge, this is the first reported case of jejunogastric intussusception following laparoscopic pancreaticoduodenectomy successfully treated via a laparoscopic approach. The minimally invasive technique proved to be safe, effective, and durable, as evidenced by the patient’s two-year recurrence-free follow-up, during which she also completed a full-term pregnancy without complications.

## Conclusions

Jejunogastric intussusception, though exceedingly rare following pancreaticoduodenectomy, should be considered in the differential diagnosis of patients with prior upper gastrointestinal surgery presenting with acute abdominal symptoms. Timely imaging and endoscopic evaluation are essential for diagnosis, given the condition’s potentially life-threatening nature if not promptly addressed. This case illustrates the successful use of laparoscopy for both diagnosis and definitive management, even in the context of altered postoperative anatomy. It supports the expanding role of minimally invasive surgery in the treatment of rare complications, offering a safe and effective alternative to open procedures when performed by experienced surgical teams.
